# Combination of focused ultrasound blood‐brain barrier opening with Lecanemab therapy in mild Alzheimer's disease

**DOI:** 10.1002/alz70856_106394

**Published:** 2026-01-08

**Authors:** Ali R Rezai, Pierre‐Francois D’Haese, Victor Finomore, Jeffrey S Carpenter, Rashi Mehta, Manish Ranjan, Camila Vieira Ligo Teixeira, Tasneem Arsiwala, Joseph Malone, Marc W Haut

**Affiliations:** ^1^ Rockefeller Neuroscience Institute, West Virginia University, Morgantown, WV, USA; ^2^ University of Pittsburgh Medical Center, Pittsburgh, PA, USA; ^3^ Rockefeller Neuroscience University, West Virginia University, Morgantown, WV, USA

## Abstract

**Background:**

Alzheimer's disease (AD) is a major neurodegenerative disorder affecting millions worldwide, with limited treatment options and significant challenges in drug delivery. Monoclonal antibody (mAb) therapies targeting β‐amyloid plaques have shown promise but are constrained by potential adverse effects, including amyloid‐related imaging abnormalities (ARIA), and extended treatment durations. Low‐Intensity Focused Ultrasound (FUS) has emerged as a promising, non‐invasive approach for targeted BBB opening. FUS‐BBB opening alone has shown small Aβ reductions, with single‐digit SUVR decreases over 9 to 16 weeks in small studies. We have now been investigating targeted mAb therapeutics in conjunction with FUS BBB opening in mild AD (NCT05469009). Our latest findings demonstrate the capability of FUS‐BBB opening to accelerate the therapeutic effects of aducanumab, resulting in an additional 32% SUVr reduction in amyloid plaque within brain regions treated with FUS+mAb compared to mAb therapy alone over 26 weeks. With the FDA approval of Lecanemab, we have expanded our clinical trial to explore the safety and efficacy of combining FUS‐BBB opening with this mAb.

**Methods:**

As the clinical trial progresses (NCT05469009), we continue recruiting amyloid‐positive Mild cognitive impairment participants, who undergo sessions of combining FUS‐BBB opening with Lecanemab. Given the ARIA risk associated with Lecanemab alone we selected patients without ARIA when treated with Lecanemab alone to ensure they could complete the full combination treatment. Neurological and cognitive assessments, along with amyloid PET scans, are performed regularly to evaluate safety, cognitive outcomes and amyloid plaque burden.

**Results:**

We have successfully recruited and treated two patients with the combination of FUS‐BBB opening and Lecanemab. Up to date safety, Aβ PET results, and cognitive assessment will be presented and discussed at the meeting.

**Conclusion:**

The preliminary results suggest safety and feasibility of combining FUS‐BBB opening with Lecanemab for targeted therapy in AD. The study highlights the potential of accelerated amyloid plaque clearance with combined FUS‐BBBO and mAb. Additional studies with more patients and extended follow‐up periods are warranted to validate these initial findings, aid in patient selection, optimized mAB and FUS‐BBB doses, and evaluate the potential clinical benefits of this novel approach.

